# Defect Prevention in Selective Laser Melting Components: Compositional and Process Effects

**DOI:** 10.3390/ma12223791

**Published:** 2019-11-18

**Authors:** Hossein Eskandari Sabzi, Pedro E. J. Rivera-Díaz-del-Castillo

**Affiliations:** Department of Engineering, Lancaster University, Gillow Ave, Bailrigg, Lancaster LA1 4YW, UK; h.eskandarisabzi@lancaster.ac.uk

**Keywords:** additive manufacturing, solidification cracking, porosity, austenitic stainless steel

## Abstract

A model to predict the conditions for printability is presented. The model focuses on crack prevention, as well as on avoiding the formation of defects such as keyholes, balls and lack of fusion. Crack prevention is ensured by controlling the solidification temperature range and path, as well as via quantifying its ability to resist thermal stresses upon solidification. Defect formation prevention is ensured by controlling the melt pool geometry and by taking into consideration the melting properties. The model’s core relies on thermodynamics and physical analysis to ensure optimal printability, and in turn offers key information for alloy design and selective laser melting process control. The model is shown to describe accurately defect formation of 316L austenitic stainless steels reported in the literature.

## 1. Introduction

In additive manufacturing (AM), printability defines the ability of a feedstock to be successfully deposited on a substrate as a bulk material, avoiding significant defects, whilst acheiving desired mechanical properties [1]. Printability can be compared to the concept of weldability, which shows the possibility and easiness of two alloys to be welded. However, unlike welding technology, there is not a comprehensive methodology to define the printability of a material under different metallurgical and process conditions.

Powder bed fusion (PBF) methods have been widely used for metal and alloy printing [2]. PBF processes can be divided into selective laser melting (SLM) and electron beam melting (EBM). A group of engineering alloys widely used in different industries is stainless steels. They can be categorised into four main groups based on their microstructures: ferritic, martensitic and precipitation hardening (marageing), duplex and austenitic [3]. Here we focus on the printability of austenitic stainless steels such as 316L due to their wide application in industry and their susceptibility to microcrack and porosity formation during SLM.

SLM of austenitic stainless steels can be performed under a wide variety of process parameters including laser power (68–400 W), scan speed (0.3–2.5 m/s), layer thickness (20–50 μm), and hatch distance; the distance between the centre of two adjacent melt pools (40–140 μm) [4,5,6,7,8,9,10,11,12,13,14,15]. Therefore, depending on the process parameters, the alloy may experience a wide variety of temperature gradients, solidification conditions, thermal stresses, and be susceptible to defects such as cracks, pores, and residual stresses. In addition to the processing parameters, changes in the chemical composition of the powder can cause differences in solidification conditions and defect formation [16].

There are two main challenges upon the SLM of austenitic stainless steels: solidification cracking and porosity formation. In the case of solidification cracking, reports on the formation of hot cracks in SLM-produced 316L austenitic stainless steel point out the presence of impurity elements such as silicon, phosphorus, and sulphur in the cracked regions [12,17,18,19,20,21,22]. Their synergistic impact with other alloying elements can lead to significant microsegregation during solidification and cause microcracking. Reported efforts to modify the chemical composition of austenitic stainless steels have led to improvements; however, such compositional changes are mainly based on welding literature and are circumscribed to such context [23].

As for porosity formation, some attempts intended to correlate process parameters to 316L as-built part density, both in laser metal deposition (LMD) and SLM [5,24]. Philo et al. [4] studied the formation of keyholes during SLM of 316L alloy using finite element method (FEM) at the mesoscale. Recently, Mukherjee et al. [2] used mechanistic models to study the printability of 316L stainless steel with a focus on lack of fusion. However, there is no comprehensive modelling approach to predict the formation of different types of porosity for different SLM parameters in austenitic stainless steels. Conventionally, numerous studies attempted to correlate the volumetric energy (heat input), which is the ratio between laser power *P* and scan speed *v*, layer thickness *t* and hatch distance *h*, to form porosity [25,26,27]. But, this expression does not consider the effects of material properties, and also other process parameters such as laser spot size. Therefore, other criteria for the prediction of porosity formation during SLM need to be developed.

Printability has traditionally been determined by trial and error experiments, so as to identify regions of successful material deposition. This approach is effective in producing high quality parts, but ignores the complex relationships between the thermal, physical, mechanical, chemical, and metallurgical processes involved. Moreover, the optimised process parameters obtained cannot be applied to other types of alloys or SLM machines. Therefore, this approach is time consuming, costly and not amenable to scaled adoption.

Although Empirical/trial and error approach is needed, but a physics-based understanding is also needed to account for both the properties of the alloy and the physics of the SLM process. Such models can be validated by experiments. In this work, the aim is to present a theory-based approach incorporating the effects of chemical composition and process parameters on defect formation during SLM. Using this approach, many physical and metallurgical aspects can be accounted for simultaneously and the best conditions for both composition and process parameters can be determined to print a component without critical defects. The proposed approach is validated with literature results, testing the methodology presented in this paper for the printability of a defect-free austenitic stainless steel.

## 2. Printability of Austenitic Stainless Steels

The critical defects that can cause problems in printing austenitic stainless steels are solidification cracking (hot cracking) and porosity (lack of fusion, balling, keyholing) [19,28,29,30]. Both the composition of the alloy and SLM parameters can affect formation of these defects. In the next sections, each of these defects and the factors that can affect them will be discussed.

### 2.1. Solidification Cracking

**Figure 1:**
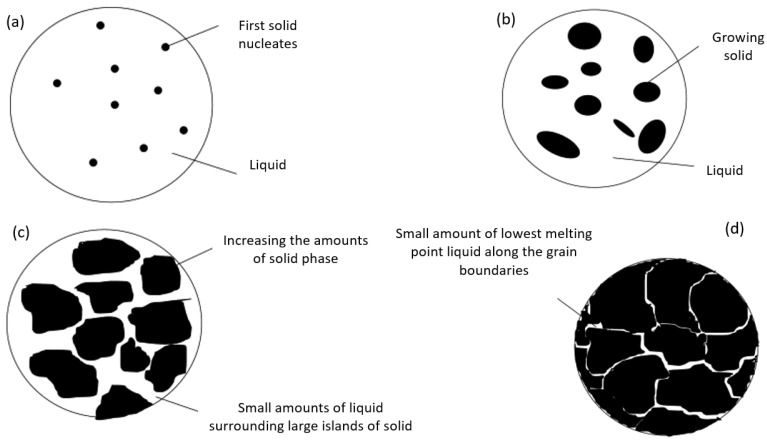
Schematic showing how an alloy solidifies. (**a**) The beginning of solidification (near liquidus temperature); (**b**) solid phase grows through the liquid phase; (**c**) the amount of solid increases by lowering temperature; (**d**) in the brittle temperature range, low amounts of liquid should remain along the grain boundaries of solid [32].

Solidification cracks can form in the melting zone due to the segregation of alloying elements. The susceptibility to solidification cracking can be quantified by defining the brittle temperature range (BTR), within which the volume fraction of the remaining liquid phase during solidification is less than 5% [31]. Figure 1 shows schematically the sequence of alloy solidification. The first solid forms from liquid and acts as a nucleus to which further atoms attach, forming a dendrite (Figure 1a). The dendrite size increases in forthcoming stages of solidification together with other dendrites until grains are formed (Figure 1b,c). Since alloys solidify over a range of of temperatures, it can be assumed that the first metal to solidify displays the highest melting point composition, whilst the latest displays the lowest melting point composition, the eutectic composition. Consequently, the lowest melting point alloy composition is pushed ahead of the solidifying dendrite, until it becomes trapped in between the grain boundaries (Figure 1d). Therefore, a large difference between the melting point of the eutectic and the bulk alloy results in an increase in solidification (hot) cracking susceptibility [32]. Therefore, the first criterion for designing crack-free austenitic stainless steels is to minimise the solidification temperature range (STR), the difference between liquidus and solidus temperatures.

In addition to STR, the unique characteristics of the SLM can also affect the cracking behaviour of the alloy. During SLM, generated thermal stresses can be measured using Equation (1) [33]:

(1) $$\sigma_{thermal}=\left[\frac{E\cdot \alpha_{CTE}}{2(1-\nu )}\right]\Delta T$$

where *E* is the Young’s modulus, $\alpha_{CTE}$ is the coefficient of thermal expansion, ν is the Poisson’s ratio, and ΔT is the temperature difference on the surface being heated. Therefore, alloys with lower $\alpha_{CTE}$ display lower thermal stresses upon SLM. Focusing on the material properties, a performance index (PI) can be defined to rank the materials compared to each other [33]:

(2) $$PI=\frac{\sigma_{ys}}{\alpha_{CTE}}$$

where $\sigma_{ys}$ is the yield strength of the material; this can be defined as [34]:

(3) $$\sigma_{ys}=\Delta \sigma_{gb}+\Delta \sigma_{ss}+\Delta \sigma_{pr}$$

where $\Delta \sigma_{gb}$ relates to the grain boundary hardening resulting from grain size, $\Delta \sigma_{ss}$ is the solid solution hardening, and $\Delta \sigma_{pr}$ is the precipitation hardening. The most important strengthening term in printing austenitic stainless steels is solid solution hardening, because precipitation is inhibited under fast cooling conditions. It can be calculated via [35]:

(4) $$\Delta \sigma_{ss}={\left[\sum_{i}\left(k_{ss,i}^{\frac{3}{2}}c_{i}\right)\right]}^{\frac{2}{3}}$$

where $k_{ss,i}$ is the solid solution strengthening coefficient for element *i* and $c_{i}$ is the volume fraction of the corresponding element *i*. Because the aim is stainless steel alloy design, only solid solution strengthening will be considered. In order to define PI, two terms of strength and coefficient of thermal expansion of the material should be calculated based on the chemical composition. $K_{ss,i}$ for different alloying elements in an austenitic matrix are presented in Table 1.

$\alpha_{CTE}$ of a material can be calculated via a linear combination of the concentration of each alloying element and its corresponding coefficient [37]. The $\alpha_{CTE}$ of different elements at temperatures near their melting points are also listed in Table 1. Maximising the PI can lead to good thermal shock resistance and a reduction in crack formation, which is the second criterion for designing a crack-free austenitic stainless steel.

**Figure 2:**
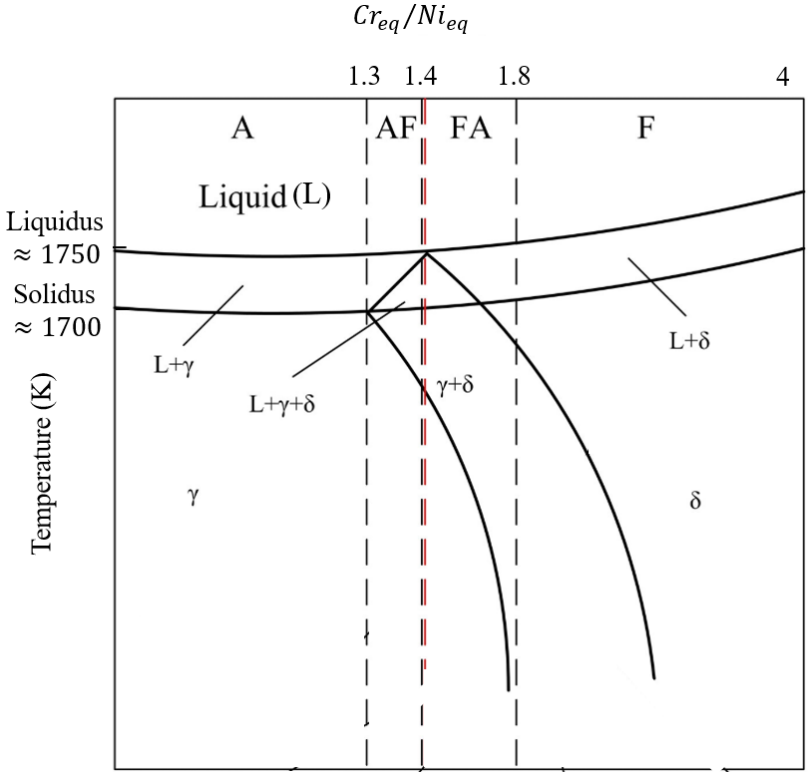
Solidification modes of austenitic stainless steels based on different $Cr_{eq}/Ni_{eq}$ values.

In welding literature, one way to assess the susceptibility of an austenitic stainless steel to cracking is the solidification mode. As in SLM the part will be built in layers, these layers should be bonded to each other. The third criterion adopted here stems from the welding literature, which can also help in reducing cracking. There are four solidification modes, based on the subsequent solid-state transformations [16] (Figure 2):

(1) Austenitic (A) (L→L+γ→γ)
(2) Austenitic-ferritic (AF) (L→L+γ→L+δ+γ→γ+δ)
(3) Ferritic-austenitic (FA) (L→L+δ→L+δ+γ→γ+δ)
(4) Ferritic (F) (L→L+δ→δ→δ+γ)

In order to predict which mode dominates during solidification, the ratio of chromium equivalent and nickel equivalent has been widely adopted, as it determines the stability of δ-ferrite and austenite upon solidification. Different relationships have been proposed to account for those values [38,39,40,41]; however, the most comprehensive has been presented by Hull [42]:

(5) $$Cr_{eq}=W_{Cr}+1.21W_{Mo}+0.48W_{Si}+0.14W_{Nb}+2.2W_{Ti}+0.72W_{W}+0.21W_{Ta}+2.27W_{V}+2.48W_{Al}$$

(6) $$Ni_{eq}=W_{Ni}+0.11W_{Mn}+24.5W_{C}+18.4W_{N}+0.44W_{Cu}+0.41W_{Co}$$

where $W_{i}$, i=Cr, Mo, Si, W, Nb, ... is the *wt*.% of element *i*.

The relationship between $Cr_{eq}/Ni_{eq}$, and solidification modes has been depicted in Figure 2. When $Cr_{eq}/Ni_{eq}$ is higher than 1.3, the presence of δ-ferrite is ensured during solidification [43], and if this ratio is higher than 1.4, the solidification mode will change from AF to FA [44]. It is widely accepted that the presence of δ-ferrite can improve solidification cracking resistance of austenitic stainless steels during welding, by reducing STR [45]. δ-ferrite has also a better ability than austenite to dissolve the impurity elements during solidification. Table 2 shows the partition coefficients of the most important impurity elements in austenitic stainless steels (Si, P, and S). Solute microsegregation increases significantly with decreasing the partiotion coefficient [46]. Therefore, these impurity elements can be dissolved in δ-ferrite, which can lead to a decrease in interdendritic segregation and a subsequent chance of solidification cracking. The presence of δ-ferrite during solidification is the basis of the third criterion for alloy design. It should be noted that δ-ferrite only is present during solidification and it will be transformed to austenite after solidification completion.

### 2.2. Porosity Formation

**Figure 3:**
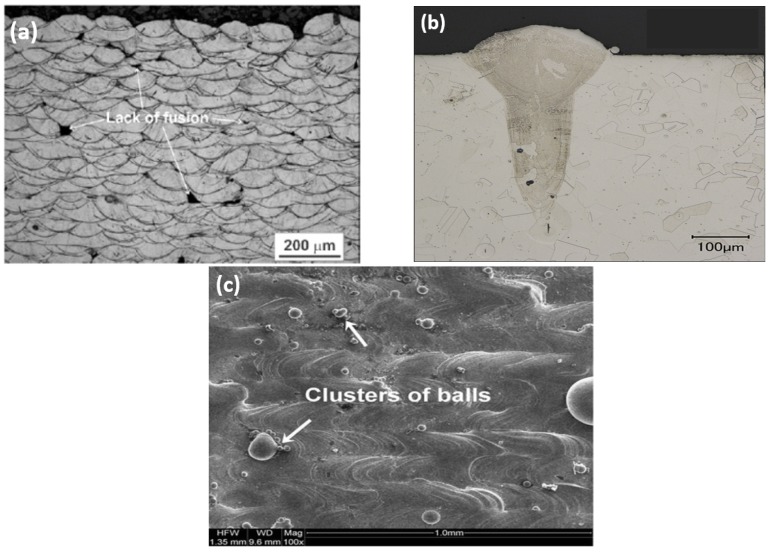
(**a**) Micrograph showing lack of fusion pores; (**b**) keyhole porosity [29]; (**c**) balling defect [52].

Porosity can be detrimental to mechanical properties. Pores can be divided into two main categories: spherical and irregular shapes. Spherical pores can be attributed to gas entrapment during powder production [47], and are thought to be acceptable at the component level, with a maximum acceptable amount in the range of 0.7% [48]. Irregularly shaped pores are divided into three types: lack of fusion, keyhole and balling. Lack of fusion pores stem from insufficient penetration of the melt pool into the previous layer (Figure 3a) [49]. The keyhole effect is mostly due to very high heat inputs that can cause vapourisation and the formation of deep V-shape melt pools as can be seen in Figure 3b [50]. Balling is a periodic change in the size and shape of the solidified track, which is due to capillarity-driven instabilities of the melt pool. This phenomenon can lead to the formation of voids and problems for even powder spreading of the subsequent layers (Figure 3c) [51].

**Figure 4:**
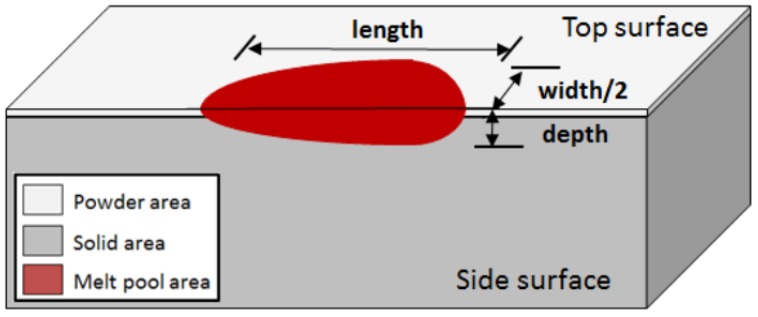
The schematics of the definition of melt pool depth (*D*), width (*W*), and length (*L*) [53].

Irregularly shaped pores directly result from SLM processing parameters. Any changes in such parameters lead to a change in the melt pool geometry. Therefore, in order to predict porosity, the melt pool geometry should be predicted. Based on available data for additive manufacturing, laser processing, and welding, some thresholds for melt pool geometry and formation of each type of porosity can be defined. A schematic of the melt pool geometry is shown in Figure 4.

The lack of fusion threshold can be determined by two criteria. Firstly, the ratio between the depth of the melt pool (*D*) and the layer thickness of the process (*t*) [49]. Secondly, the ratio between hatch distance (*h*) and the width of the melt pool (*W*) can determine the good penetration of the melt pools on each other [30]. These conditions respectively dictate lack of fusion in the laser direction and normal to it.

The formation of keyholes can be predicted in two different ways. The ratio between width and depth of the melt pool determines the possibility of keyhole formation and secondly, based on the processing parameters and thermophysical properties such as enthalpy of melting of the alloy, the threshold energy for keyholing can be calculated [29]. Both stem from excess heat per unit volume, which can cause local evaporation.

The ratio between the length (*L*) and width of the melt pool also can define a criterion for balling, as the melt pool needs to uniformly redistribute the flow behind the advancing laser [54]. Quantitative expressions for these criteria will be presented in next sections.

## 3. Methodology

### 3.1. Optimisation of Chemical Composition for Crack Prevention

Three composition-dependent functions should be optimised. STR should be minimised, PI should be maximised, and $Cr_{eq}/Ni_{eq}$ needs to be higher than 1.3, changing the chemical composition of the alloy.

### 3.2. Porosity Formation Prevention Criteria

For lack of fusion prevention, melt pool geometry must meet two semi-empirical conditions: D/t>1.5 and h/W<1 [30,49]. For balling prevention, the criterion is L/W<2.3 [54]. For keyholing prevention two approaches exist. First criterion is W/D>2. The second is based on the processing parameters and physical properties of the material. Using the concept of normalised enthalpy, the energy needed to avoid vapourisation and the formation of keyholes can be estimated [29]. The normalised enthalpy can be calculated via the following equation:

(7) $$\frac{\Delta H}{h_{s}}=\frac{AP}{h_{s}\sqrt{\pi dv\sigma^{3}}}$$

where *A* is the powder absorptivity, *P* is the laser power, $h_{s}$ is the enthalpy of melting, *d* is the thermal diffusivity, *v* is the laser scan speed, and σ is the laser spot size (the area covered by the laser). An estimate for keyhole threshold in terms of normalised enthalpy is $\frac{\Delta H}{h_{s}}>\frac{\pi T_{b}}{T_{m}}$[29]. $T_{b}$ and $T_{m}$ are the boiling and melting temperatures, respectively. Using values for melting and vapourisation for austenitic stainless show that the threshold for keyhole formation in austenitic stainless steels is about 7.

To summarise, the defects listed above and their prevention means are presented in Table 3. Except from the balling prevention criterion, the same as reported in the literature, all the other criteria have been modified and compared with their origin. For the first time, criteria to avoid microcracking in SLM of austenitic stainless steels are presented. Conditions for lack of fusion, the criteria reported in literature have been modified to two distinct and simple criteria. For keyhole prevention, the appropriate use of the normalised enthalpy and the correct threshold have been presented here.

#### Model for Melt Pool Dimensions

In order to predict the melt pool geometry based on different processing parameters, a combination of physical models which can relate the material properties to SLM processing parameters has been used.

The width of the melt pool (*W*) can be described by [56]:

(8) $$W=\sqrt{\frac{8}{\pi e}\frac{AP}{\rho C_{p}v\left(T_{m}-T_{0}\right)}}$$

where ρ is the density, $C_{p}$ is the heat capacity, and $T_{0}$ is the initial temperature of the powder bed.

For the melt pool depth (*D*) and length (*L*), the Eagar-Tsai model is a good estimation [57]. Firstly two dimensionless parameters of *F* and *B* should be introduced. *F* can be defined as the ratio between the laser dwell time and thermal diffusion time, which is:

(9) $$F=\sqrt{\frac{d}{v\sigma }}$$

The second dimensionless parameter (*B*) is the ratio between laser deposited energy density and enthalpy of melting, which can be simplified as:

(10) $$B=\frac{\Delta H}{2^{3/4}\pi h_{s}}$$

Both *D* and *L* are functions of *B* and *F*. The expressions that can be used for *D* and *L* predictions can be simplified into these final equations:

(11) $$\begin{array}{ll} D & =\frac{\sigma }{\sqrt{F}}[0.008-0.0048B-0.047F-0.099BF+\left(0.32+0.015B\right)F\;\ln F\\ & +\ln B(0.0056-0.89F+0.29F\;\ln F)] \end{array}$$

(12) $$L=\frac{\sigma }{F^{2}}\left[0.0053-0.21F+1.3F^{2}+\left(-0.11-0.17B\right)F^{2}\;\ln F+B\left(-0.0062+0.23F+0.75F^{2}\right)\right]$$

The values for *B* and *F* are only valid for the materials that have F<1, such as austenitic stainless steels. For materials which have F>1, *B* and *F* should be measured in other ways discussed in [57], which is not the subject of this paper.

## 4. Model Validation

Experimental data on SLM of 316L stainless steel is presented next to relate chemical compositions and process parameters reported in the literature to different types of defects. The criteria for defect prevention are compared to the exisiting data in the literature, and their reliability is shown.

### 4.1. Cracking Behaviour of 316L Stainless Steel

As 316L alloy is an industrially relevant austenitic stainless steel due to its wide application, here we focus on the cracking behaviour of this type of steel. Table 4 lists different compositions of 316L stainless steel, which have been produced using SLM; in the first three printed parts (shown in bold) microcrack formation has been reported. STR, PI, and $Cr_{eq}/Ni_{eq}$ are also calculated for each of these alloys. The first alloy in this list has an unappropriate solidification mode (A mode), the second alloy has high STR of 60 K, and the third alloy has a low PI of 1.19 × 10^6^ MPa·K.

In order to reduce the cracking susceptibility, alterations in alloy composition of 316L stainless steel can be performed. Table 4 also shows 11 different variations of 316L compositions that have been printed successfully without microcracks being reported. All of these alloys solidify in FA mode. Furthermore, there is a compromise between STR, PI and $Cr_{eq}/Ni_{eq}$ in all cases. When STR is lower than 39 K, the alloys show a safe solidification behaviour, no matter what is their PI and $Cr_{eq}/Ni_{eq}$. However, when STR is equal or higher than 39 K, the situation gets more complicated. For the alloys printed in [5,58], high PI compensates the wide solidification temperature range. For the three last alloys listed in Table 4, the low PI will be compensated by high $Cr_{eq}/Ni_{eq}$ values, which is indicative of higher δ-ferrite during solidification. The minimum STR and maximum PI that have been reported in the literature is 32 K and 1.46 × 10^6^ MPa·K, respectively, which can be used as metrics for designing new alloy formulations.

### 4.2. Porosity and Defect Formation Behaviour of 316L Stainless Steel

**Figure 5:**
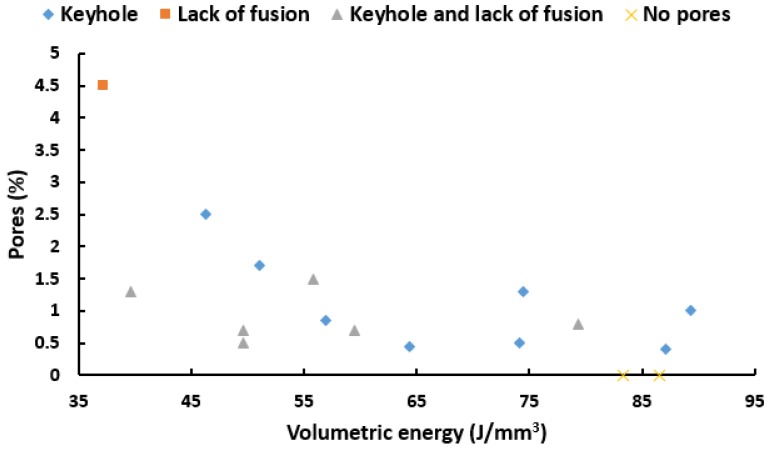
Porosity map that shows the experimental porosity percentage for some of the alloys presented in Table 4, based on the volumetric energy. The predictions of the proposed model in this paper are also included in this diagram.

As processing parameters are the most important factor that can affect the formation of porosity during SLM, several attempts have been made on their optimisation to maximise part densities. Table 5 shows the different combinations of SLM processing parameters and their reported melt pool dimensions as well as the predicted porosity types based on the criteria presented in this paper, and the real observations. The normalised enthalpy for each of the process parameter sets is calculated whenever the alloy composition and SLM machine parameters are reported. The criteria mentioned in 3.2 not only can predict the formation of pores based on the melt pool dimensions, but also the exact types of defects after manufacturing. In all cases that the model predicts formation of specific pores, the percentage of pores is more that 0.1%. For alloy number 3, where no pores are predicted by the criteria presented here, a perfect build has been reported. The normalised enthalpy values for alloy 3 is below the critical value of 7, which shows the reliability of using this concept for keyhole prevention. The alloys with normalised enthalpies higher than 7 underwent keyhole formation. Occurence of lack of fusion and balling were also as expected based on the criteria mentioned before in this paper. In order to show the inability for the conventional methods to predict porosity, the volumetric energy ($\frac{P}{v.h.t}$) has been plotted for each of full-reported sets of experiments in Table 5 and shown in Figure 5. It is seen that volumetric energy cannot be correlated with pore formation. The reason is that in addition to laser power, scan speed, layer thickness, and hatch distance, the laser spot size should be considered as an important variable of SLM that can affect the density of the as-built part. Moreover, the volumetric energy cannot consider the effects of material properties on the formation of pores.

**Figure 6:**
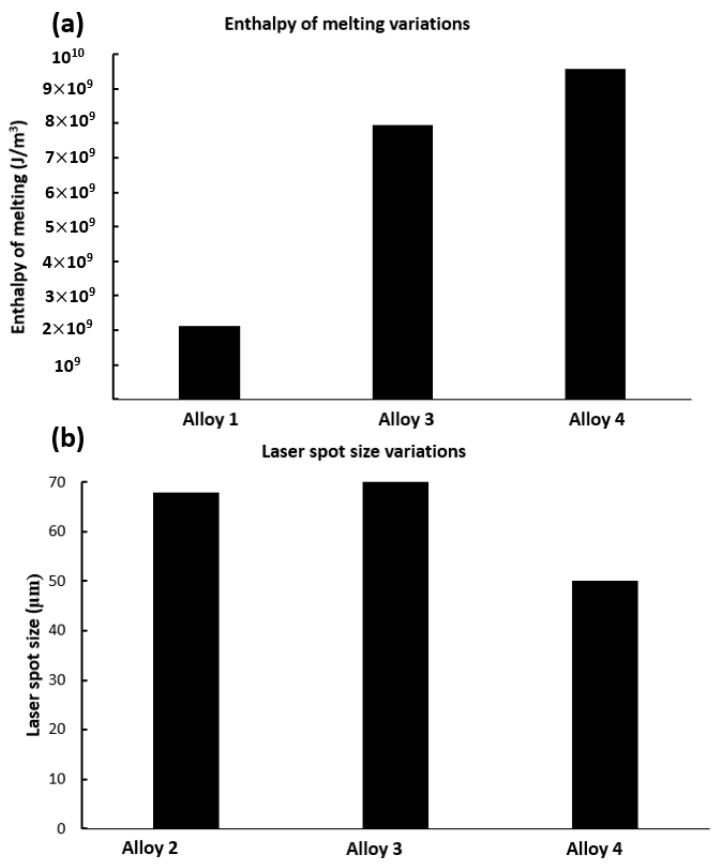
(**a**) Huge differences of the enthalpy of melting of the alloys 1, 3, and 4 of the Table 5. (**b**) Different laser spot sizes that have been used to build the alloys 2, 3, and 4 of the Table 4.

Based on the model presented in this paper for porosity prevention, the laser spot size and the enthalpy of melting of the alloy are amongst the two most important parameters that can affect porosity and defects. Figure 6 shows the variations of these two values based on a change in chemical composition of the alloy and the SLM machines (for alloys number 1–4 of Table 5). Even small changes in chemical composition can cause large differences in enthalpy of melting of the alloy and the resulting pore percentage, which has been ignored in conventional approaches for porosity prediction. Moreover, laser spot size variations can significantly affect the keyhole formation (by changing the normalised enthalpy values) and melt pool depth and length. Therefore, instead of the conventional approach to estimate the part density after SLM, the methodology presented here can lead to a precise estimation about the density of the SLM as-built parts.

Some works have tried to simulate the melt pool geometry of SLM-produced 316L stainless steel, using finite element methods [2,68]. However, because of the complexity of heat transfer phenomena and the differences between physical properties of the powder and the bulk material, the FEM simulations suffer from precision. Furthermore, these methods are expensive and time consuming. Most of finite element simulations set some of the SLM parameters as constants and try to simulate the process. Although the new methodology presented in this paper assumes many simplifications and some of the heat transfer phenomena are neglected, but it can predict melt pool geometry in an economic and time consuming manner and can provide part producers a good overview for choosing the right processing parameters whilst accounting for compositional variations.

## 5. Summary and Conclusions

A novel methodology has been presented to predict the formation of two most important issues during selective laser melting of austenitic stainless steels: solidification cracking and porosity formation. In the case of solidification cracking, three functions of solidification temperature range (difference between liquidus and solidus temperature), the performance index (ratio between the yield strength and coefficient of thermal expansion of the material), and $Cr_{eq}/Ni_{eq}$ (determining the solidification path) have been presented as metrics to predict the possibility of cracking. The minimisation of the solidification temperature range, the maximisation of performance index, and the presence of δ-ferrite during solidification predict the minimisation of solidification cracking susceptibility. The criteria defined in this paper have been compared to existing data in literature for selective laser melted 316L stainless steel.

In the case of porosity formation, different criteria which correlate SLM processing parameters and the physical properties of the material with the quality of formation of melt pool and solidification behaviour have been defined. A novel physical model has been suggested to predict the conditions for keyhole formation and also the melt pool geometry. The proposed criteria have been compared with literature data on SLM of 316L alloy showing remarkable agreement.

## Figures and Tables

**Table 1:** Coefficients for solid solution hardening for different elements (*i*) in austenitic matrix [35], and their thermal expansion coefficient near their melting point [36].

*i*	Ni	Cr	Mo	W	C	N	Si	Fe (FCC)
$k_{ss,i}$ [MPa at%^−3/2^]	112	101.71	637	826	1984	1984	-	-
$\alpha_{CTE}$ [10^−6^ K^−1^]	20.3	19	16.5	11.6	-	-	3.8	23.3

**Table 2:** Partition coefficient of the most important impurity elements in austenite and δ-ferrite [23].

Impurity Element	Partition Coefficient in γ	Partition Coefficient in δ
Sulphur	0.035	0.091
Phosphorus	0.13	0.23
Silicon	0.52	0.77

**Table 3:** The summary of the defects appearing during SLM and the criteria proposed in this study for avoiding them. The references and how this paper modified the criteria are also provided.

Phenomenon	Criteria	Ref.	Notes
Microcracks	STR minimisation		Imposed to reduce formation of low melting point eutectics
	PI maximisation		Defined as thermal shock resistance
	Presence of *δ*-ferrite		Alleviating detrimental effects
	during solidification		of impurity elements
Lack of fusion	D/t>1.5	[30]	A ratio of 1.1 has been suggested by Mukherjee et al.
	h/W<1	[49]	Tang et al. claimed the criteria for lack of fusion
			prevention is ${\left(h/W\right)}^{2}+{\left(t/D\right)}^{2}<1$
Keyholes	W/D>2	[55]	Johnson et al. suggested a ratio of 1.5 rather than 2
	$\Delta H/h_{s}<7$	[29]	King et al. suggested a threshold of 30 rather than 7
Balling	L/W<2.3	[54]	-

**Table 4:** Chemical compositions of 316L stainless steel produced by SLM. The units of STR and PI are K and MPa·K, respectively. Fe contents are balanced and not shown in the table. The alloys in bold font underwent cracking.

Cr	Ni	Mn	Mo	C	N	Si	P	S	STR	PI $\times 10^{6}$	${Cr}_{eq}/{Ni}_{eq}$	Ref.
**16.17**	**12.57**	**0.23**	**2.33**	**0.098**	**-**	**0.6**	**0.014**	**0.014**	**50**	**1.32**	**1.28**	[17]
**20.7**	**11.4**	**1.32**	**2.45**	**0.02**	**0.09**	**0.5**	**0.02**	**0.01**	**60**	**1.46**	**1.58**	[19]
**17.26**	**11.48**	**1.41**	**2.32**	**0.018**	**-**	**0.71**	**0.01**	**0.01**	**39**	**1.19**	**1.68**	[12]
17.34	10.74	1.14	2.28	0.01	0.1	0.63	0.026	0.014	43	1.36	1.57	[5]
17.5	11.5	2	2.25	0.03	0.11	1	0.045	0.03	57	1.45	1.54	[58]
17.42	12.53	0.6	2.36	0.02	0.06	0.51	0.01	0.01	32	1.36	1.45	[8]
17	12	1	2.5	0.015	0.05	0.5	0.023	0.01	38	1.35	1.51	[59]
17.75	12.75	1.5	2.4	0.02	-	-	0.01	0.001	33	1.24	1.49	[10]
16.7	11.9	0.6	2.5	0.02	-	0.6	0.01	0.02	33	1.22	1.61	[11]
16.7	10.3	0.99	2.2	0.01	-	0.69	0.02	0.05	37	1.12	1.85	[60]
17.9	12.8	1.15	2.35	0.018	0.09	0.66	0.01	0.004	33	1.42	1.4	[61]
17.5	11.2	2.2	2.3	0.03	-	-	0.05	0.03	61	1.22	1.67	[62]
16.3	10.3	1.31	2.09	0.026	-	0.49	0.026	0.006	44	1.14	1.72	[14]
18.43	12.2	1.86	2.46	0.02	-	0.75	0.032	0.01	54	1.26	1.69	[15]

**Table 5:** SLM processing parameters including *P* (W), *v* (mm/s), *t* (μm), and *h* (μm) that have been used for production of 316L stainless steel in four different studies. For each set of experiments the measured melt pool dimensions *L* (μm), *W* (μm), and *D* (μm) (or some of them) and their related normalised enthalpies have been presented. The variables represented by dashes, have not been reported in the original reference. In cases where the laser spot size or the full composition were not reported, $\Delta H/h_{s}$ could not be calculated and N/A is shown. The prediction about lack of fusion (LOF), keyhole formation (K), balling (B) and experimental measurements are also presented.

Alloy	*P*	*V*	*t*	*h*	*L*	*W*	*D*	$\Delta H/h_{s}$	Prediction	Pores	Ref.
1	200	850	30	90	-	146	110	N/A	K	0.4 %	[63]
1	200	1000	30	90	-	130	85	N/A	K	0.5 %	[63]
1	200	1150	30	90	-	104	85	N/A	K	0.45 %	[63]
1	200	1300	30	90	-	104	70	N/A	K	0.85 %	[63]
1	200	1450	30	90	-	104	75	N/A	K	1.7 %	[63]
1	200	1600	30	90	-	100	70	N/A	K	2.5 %	[63]
2	100	100	50	40	-	180	30	N/A	LOF	Not reported	[64]
2	100	200	50	40	-	140	20	N/A	LOF	Not reported	[64]
2	100	300	50	40	-	120	10	N/A	LOF	Not reported	[64]
2	100	400	50	40	-	110	5	N/A	LOF	Not reported	[64]
3	175	500	30	140	-	175	75	6.9	No pores	No pores	[65]
3	400	1100	30	140	-	250	110	4.3	No pores	No pores	[65]
4	250	1500	-	-	526	105.6	52.8	41.8	B	0.9 %	[66]
4	250	1800	-	-	523.5	100.8	48	38.2	B	1.39 %	[66]
4	400	1800	-	-	846.4	129.6	60	61.1	B	1.2 %	[66]
4	100	400	-	-	212.4	129.6	59.5	32.4	No B/K	0.93 %	[66]
5	400	1800	30	112	32	112	105	8.7	K	0.5 %	[67]
5	400	1500	30	112	79	103	119	9.4	LOF/K	0.8 %	[67]
5	300	1800	30	112	57	94	65	7.1	LOF/K	0.7 %	[67]
5	300	1500	30	112	35	83	94	7.5	LOF/K	0.7 %	[67]
5	200	1500	30	112	26	84	57	6.9	LOF/K	1.3 %	[67]
5	200	1200	30	112	45	104	68	7.1	LOF/K	0.5 %	[67]
5	200	800	30	112	24	123	116	7.3	K	1.3 %	[67]
5	150	1200	30	112	21	79	30	5.12	LOF	4.5 %	[67]
5	150	800	30	112	44	109	67	6.8	LOF/K	1.5 %	[67]
5	150	500	30	112	40	115	120	7.8	K	1 %	[67]
